# Freehand Thoracic Pedicle Screw Placement: Review of Existing Strategies and a Step-by-Step Guide Using Uniform Landmarks for All Levels

**DOI:** 10.7759/cureus.501

**Published:** 2016-02-19

**Authors:** Mauricio J Avila, Ali A Baaj

**Affiliations:** 1 Neurological Surgery, Weill Cornell Medical College; 2 Neurological Surgery, NewYork-Presbyterian/Weill Cornell Medical College

**Keywords:** thoracic screws, pedicle screws, spinal fusion, thoracic vertebrae, spine deformity, spine surgery, spinal instrumentation, freehand technique

## Abstract

Pedicle screw fixation in the thoracic spine presents certain challenges due to the critical regional neurovascular anatomy as well as the narrow pedicular corridor that typically exists. With increased awareness of the dangers of intraoperative radiation, the ability to place pedicle screws with anatomic landmarks alone is paramount. In this study, we reviewed the literature from 1990 to 2015 for studies that included freehand pedicle screw placement in the thoracic spine with special emphasis on entry points and the trajectories of the screws. We excluded studies that used fluoroscopy guidance, navigation techniques, cadaveric and biomechanical articles, case reports, and experimental studies on animals. The search retrieved 40 articles, and after careful selection, seven articles were analyzed. Over 8,000 screws were placed in the different studies. The mean accuracy for placement of the thoracic screws was 93.3%. However, there is little consensus between studies in entry points, sagittal, and axial trajectories of the screws.

We complete this review by presenting our step-by-step technique for the placement of freehand pedicle screws in the thoracic spine.

## Introduction and background

Pedicle screw and rod constructs have become widely accepted as the ideal option to provide stable spinal fixation [1-8].

Traditionally, freehand pedicle screw placement in the lumbar spine has had wide acceptance; however, pedicle screw placement in the thoracic spine has posed more challenges due to more challenging anatomy [9-10].

A variety of different techniques is currently used to aid the surgeon for accurate placement of pedicle screws in the thoracic spine. These techniques include intraoperative C-arm fluoroscopy, intraoperative computer tomography (CT), and computer-assisted navigation [11-16]. Although these adjunct technologies are playing a greater role in complex deformity surgery, drawbacks include radiation exposure to the patient and surgeon as well as high cost and prolonged operative time [13, 17-20].

Freehand pedicle screw placement in the thoracic spine is commonly performed by many surgeons and is both safe and effective [1-2, 17, 21-23]. Existing techniques, however, primarily rely on surgeon preferences and biases and may not provide easily reproducible parameters. Furthermore, most techniques describe varying entry points and trajectories depending on the thoracic spinal level or region [19, 24-25]. Due to the size of the thoracic pedicles and the proximity of vital structures in this region, the placement of freehand screws requires a high level of precision [10].

The goal of this study was to comprehensively review the existing published literature on freehand thoracic pedicle screw placement techniques, focusing on similarities and differences. Additionally, we present our step-by-step technique, which relies on a uniform entry point and sagittal trajectory for all levels.

## Review

### Materials and Methods

A review of the literature using the National Library of Medicine via the PubMed database was performed from 1990 to August 2015. Search words included: “freehand”, “thoracic pedicle screw”, and “technique”. Only articles written in English were included. The title and abstract were screened and a first selection was done to include only the papers that discussed the specific techniques in the analysis. Specifically, we collected data on the number of patients, the number of inserted pedicle screws, and parameters of the surgical technique (entry point, axial, and sagittal trajectories). We excluded studies that involved fluoroscopy guidance, navigation techniques, cadaveric and biomechanical articles, case reports, and experimental studies on animals.

In order to encompass as many relevant studies as possible, we also performed a manual search of the references of selected articles.

### Results

The search yielded a total of 40 published articles. Following the first selection by title and abstract, we identified seven [1-4, 23, 26-27] eligible studies that precisely detailed the technique of freehand thoracic pedicle screws placement. Overall, the seven studies represent a total of 1,602 patients with diverse pathologies and 8,586 screws in the thoracic spine. All the studies were retrospective case series; five out of seven represented a single surgeon experience, one study represented the experience of two surgeons [2], and one study represented the experience of eight different surgeons [3]. All the studies used postoperative CT scans to verify the positioning of the screws in the thoracic spine.

In Table 1, we present the details of the seven final articles included.

**Table 1:** Studies Describing Techniques for Freehand Placement of Thoracic Pedicle Screws

Authors & Year	Patients	No. of Screws	Spine Pathology	Entry Point	Axial Trajectory	Sagittal Trajectory
Kim, et al. (2004)	394	3,204	Scoliosis (273 patients), Kyphosis (53 patients), Fracture (45 patients), Tumor (12 patients), Infection (4 patients), Failed back surgery syndrome (7 patients)	T1-T2: junction of the transverse process and lamina at the lateral pars interarticularis; T3-T6: getting more lateral and caudal; T7-T9: junction of proximal edge of the transverse process and lamina just lateral to the midportion of the base of the superior articular process; T11-T12: junction of the transverse process and lamina or just medial to the lateral aspect of the pars interarticularis.	Proximal thoracic region: more lateral and caudal. Apical mid-thoracic region: more medial and cephalad	Proximal thoracic region: more lateral and caudal. Apical mid-thoracic region: more medial and cephalad
Karapinar, et al. (2008)	98	297	Trauma (79 cases), Scoliosis (12 cases), Metastatic disease (3 cases), Degenerative spine (2 cases) Pott's disease (2 cases)	T10, T11, and T12: The junction of a vertical line along the lateral pars boundary and a transverse line dividing the transverse process in half.	“Medial orientation of the awl’s trajectory corresponded to a line drawn from the intended starting point as described to a point in the anterior vertebral body that allowed for maximum screw length and triangulation without a medial breach of the pedicle.”	No guidelines for sagittal trajectories
Modi, et al. (2009)	43	854	Scoliosis (Cobb angle < 90° ): 22 idiopathic and 21 neuromuscular scoliosis patients	The junction of the outer third and inner two-thirds of the superior facet joint taken at the junction of the lateral and medial thirds of the facet joint after observing the whole facet joint margin.	No guidelines for axial trajectories	No guidelines for sagittal trajectories
Modi, et al. (2010)	26	482	Severe scoliotic deformities (Cobb angle >90°). Five patients with adolescent idiopathic scoliosis and 21 patients with neuromuscular scoliosis	The junction of the outer third and inner two-thirds of the superior facet joint taken at the junction of the lateral and medial thirds of the facet joint after observing the whole facet joint margin.	No guidelines for axial trajectories	No guidelines for sagittal trajectories
Parker, et al. (2011)	964	3,443	Degenerative/deformity disease (51.2%), spondylolisthesis (23.7%), tumor (22.7%), trauma (11.3%), infection (7.6%), and congenital (0.9%). Total of patients for thoracic and lumbar freehand screws.	The center of a triangular bony confluence formed by the superior articular facet, the transverse process, and the pars interarticularis. Occurs medial to the lateral margin of the superior articular process.	Medio-lateral trajectory is performed to triangulate the screw insertion from lateral to medial.	Rostro-caudal trajectory parallels the superior endplate of the segment of interest.
Rivkin, et al. (2014)	44	87	“Various pathologies that needed cervicothoracic fusion” (non-described)	T1 only: medial and superior to the intersection of the transverse process and pars interarticularis.	Medial-lateral trajectory: line drawn from the tip of the spinous process to the contralateral entry point.	Cranial-caudal trajectory: perpendicular to the long axis of the T1 lamina
Fennell, et al. (2014)	33	219	61% Trauma, 18% Tumor, 12% Infection, 9% Deformity	For each level: 3 mm caudal to the junction of the transverse process and the lateral margin of the superior articulating process	Approximately 30° at T1 and T2, and 20° from T3 to T12	Always orthogonal to the dorsal curvature of the spine at corresponding level.
Total	1,602	8,586				

Spine Pathologies

There were several pathologies involved in the placement of freehand thoracic screws: from moderate scoliosis (idiopathic and neuromuscular scoliosis) to severe scoliosis (Cobb angle > 90), to infection, Mal de Pott, trauma, tumors, metastasis, and congenital problems (Table 1) [1-4, 23, 26-27].

Entry Points

Various entry points have been proposed that are mainly based on the level of the thoracic spine.

Six of the seven studies focused on several thoracic levels. Only one study [4] focused on just one vertebral level, T1, with a modification of the technique described by Kim, et al. [2]. The details of the different entry points are available in Table 1.

Trajectories

Six studies evaluated axial and sagittal trajectories [1-4, 23, 28]. Nonetheless, the degree of explanation and details for these trajectories vary greatly. The majority of the reviewed studies mentioned directions of the screw rather than actual angles on the vertical or horizontal planes. The study by Fennell, et al. mentioned an axial trajectory of 30° at the T1 and T2 vertebrae and 20° from T3 to T12 [1]. Others studies compared the trajectory of the screw to the initial entry point line. Rivkin, et al. described a horizontal plane that was parallel to a line drawn from the tip of the spinous process to the contralateral entry point [4]. One study suggested the sagittal trajectory should rely on the superior endplate visualization of a corresponding thoracic vertebra [3].

Accuracy of Freehand Thoracic Pedicle Screws

The accuracy of thoracic pedicle screws placement was reported in all the studies. In general, pedicle screw accuracy was defined as “having the entire screw contained within the cortices of each respective pedicle” [3].

Mean accuracy rate for placement of pedicle screws in the studies was 93.34% (standard deviation of 3.54). The lowest reported accuracy was 87.4% [4] in screws only at T1 and the highest was 98.3% in their series of 964 patients [3]. Modi, et al. calculated the difference in accuracy for spine pathologies and found that the accuracy for patients with adolescent idiopathic scoliosis was 86.1%, patients with cerebral palsy - 91.7%, Duchenne’s muscular dystrophy - 95.9%, spinal muscular atrophy - 90.2%, and polio - 84.4%. The differences between diseases were not statistically significantly [26].

In studies with multiple surgeons, the accuracy for five surgeons was 93.8% [2] and for eight surgeons was 98.3% [3].

Breach of the Vertebral Bodies

There was a consensus in all the studies of a “safe zone” from 2 to 4 mm for pedicle screw breach of the vertebral bodies. This safe zone allows the medial or lateral wall breach by the screw without clinical consequences for the patient [2, 23, 28]. Furthermore, the study by Kim, et al. defined a breach of < 2 mm as a “definite safe zone”, a breach of 2 to 4 mm a “probable safe zone”, and a breach of 4 to 8 mm as a “questionable safe zone” [2]. Furthermore, as shown by Karapinar, et al., the majority of the breaches are minor (< 2 mm) in the thoracic spine [23].

Overall, a lateral breach was more common than a medial breach of the vertebral bodies. There was a range of 2.5% to 21.6% of the screws for lateral breach and 1.7% to 13.2% of the screws for medial breach, with the majority falling below 5% of the screws [1-4, 23, 26-27].

Four studies reported the most common location of the vertebral bodies’ breaches. Parker, et al. [3] reported that T4 and T6 were the two most common sites for a breach with 4.1% and 4% of the screws at those levels, respectively. In the thoracolumbar junction, Karapinar, et al. [23] reported the breach rate of 16.4% at T10, 12.1% at T11, and 4.2% at T12. The two studies by Modi, et al. [26-27] reported that the middle thoracic spine (T5-T8) was the most common site for a breach in comparison with the upper and lower thoracic spine. Of the seven studies, only one reported the need for a revision surgery after a vertebral body breach in a patient with a T4 medial breach and osteomyelitis [3].

Complications

Overall, the complication rate after freehand pedicle screw placement in the thoracic spine was low. Parker, et al. reported 4.3% incidence of durotomies in their case series, however, this includes thoracic and lumbar freehand screw placement [3]. Kim, et al. reported no neurological or vascular complications in their series of 3,204 screws, which had up to 10 years follow-up [2].

### Discussion

Thoracic pedicle screw fixation can be challenging due to the complex morphology of the thoracic vertebrae, which can lead to screw malposition with potential injury to adjacent structures [2, 6, 14, 29]. Although various intraoperative navigational techniques can be helpful to avoid misplacement of the screws, radiation exposure to both the patient and the surgeon is of great concern. To avoid harmful effects of radiation, freehand pedicle screw placement has become the preferred modality of fixation for various thoracic pathologies, such as trauma, degenerative spine disease, scoliosis, and tumors [1-3, 17, 29-30]. The diversity of spine pathologies where the freehand placement of thoracic screws has been successful is encouraging. Moreover, as navigation and assisted technologies become widely available, it is crucial that the spine surgeon still masters the anatomical basis of spine instrumentation and be comfortable with freehand techniques, especially to avoid the harmful effects of sustained radiation [31-34].

The accuracy of thoracic pedicle screw placement is high. In this review, Parker, et al. reported the highest rate of accurate placement (98.3%) [3]. Their 1.7% breach rate occurred in 9% of the total cohort of their patients. Nonetheless, only 0.8% required revision surgery for misplaced screws, which furthers add to the safety of a freehand technique. It is important to clarify that some studies reported different accuracy rates depending on whether or not they considered the “safe zone” to be an “accurate” screw. For example, Modi, et al. had an accuracy rate of 65.2% without including the “safe zone”, but because a lot of the breaches were inside the “safe zone”, their reported final accuracy rate was 90.7% [26]. Interestingly, they did not find any statistically significant difference between patient diagnosis and accuracy of the placement of pedicle screws. The latter adds support to the usefulness of a freehand technique in different pathologies.

In this review, we found a high range of reported lateral breaches of the vertebral bodies. The highest reported was by Modi, et al. of 21.6% and may be due to the fact that these screws were placed in patients with severe scoliosis where the anatomical landmarks may be more difficult to delineate [26]. Nonetheless, they did not report any neurological or vascular complications despite their high percentage of a breach. Furthermore, a 2.5% lateral breach in the series by Parker, et al. [3], the 4.1% by Fennell, et al. [1], the 5.8% by Karapinar, et al. [23], and the 6.2% by Kim, et al. [2] add support to the safety and feasibility of freehand thoracic screw placement in the thoracic spine. Parker, et al. were able to compare the breach rate of the thoracic and the lumbar freehand screws; the thoracic spine had a higher rate than the lumbar (2.5% vs. 0.9%) [3]. This comes without a surprise as the pedicles in the lumbar spine are wider than the ones in the thoracic spine. The common connection between all the studies is that a lateral breach is far more prevalent than a medial breach. As Parker, et al. discussed, this may be because the surgeon will try to avoid the medial wall to prevent spinal cord damage as well as the higher thickness of the medial wall of the pedicle [3].

Kim, et al. suggested a “safe zone” for vertebral bodies’ breach (medial and lateral) as follows: breach of < 2 mm as a “definite safe zone”, a breach of 2 to 4 mm as a “probable safe zone”, and a breach of 4 to 8 mm as a “questionable safe zone” [2]. Belmont, et al. expanded the “safe zone” for lateral breach up to 6 mm [28]. However, all the studies included in this review concurred that a breach of 2 to 4 mm is safe, and there is no need for repositioning the screws if the patient is asymptomatic; for this reason, we caution the reader against using the “expanded” safe zone as the majority of the studies limit the safe zone from 2 to 4 mm. The study by Kim, et al. made the 4-8 mm limit as "questionable safe" rather than "safe" or "probably safe" [2]. We believe that the 4 mm limit is the most commonly used (and reported) upper limit for a breach. The existence of a “safe zone” for pedicle screw breach allows the spinal surgeons to confidently use a freehand technique while allowing some room for improvement.

The complication rate in the reviewed studies was low. The majority of the studies reported no neurological or vascular complications following freehand placement of thoracic screws. The study by Kim, et al. provides the strongest evidence of the safety of this technique; they have the longest follow-up (10 years) without any neurological or vascular complication reported [2]. Moreover, even when comparing eight different surgeons, Parker, et al. [3] found an overall low complication rate in the over 3,000 screws placed. The latter suggests that the technique is still safe when used by different surgeons.

The results of this review highlight the fact that, while freehand thoracic screw placement techniques may be widely employed and are common, only a handful of published studies delineate the nuances of these techniques. Furthermore, the published studies highlight various starting points and/or trajectories for each level of the thoracic spine, making the adoption of the technique cumbersome to the spinal surgeon or trainee.

### Step-by-step technique

We have recently proposed a technique that relies on less variable parameters and may be easier to teach [1]. We have had excellent early clinical experience with this at major training institutions and the clinical case series has previously been examined. Here, for the first time, we highlight a more detailed step-by-step guide to our technique:

Step 1

**Figure 1:**
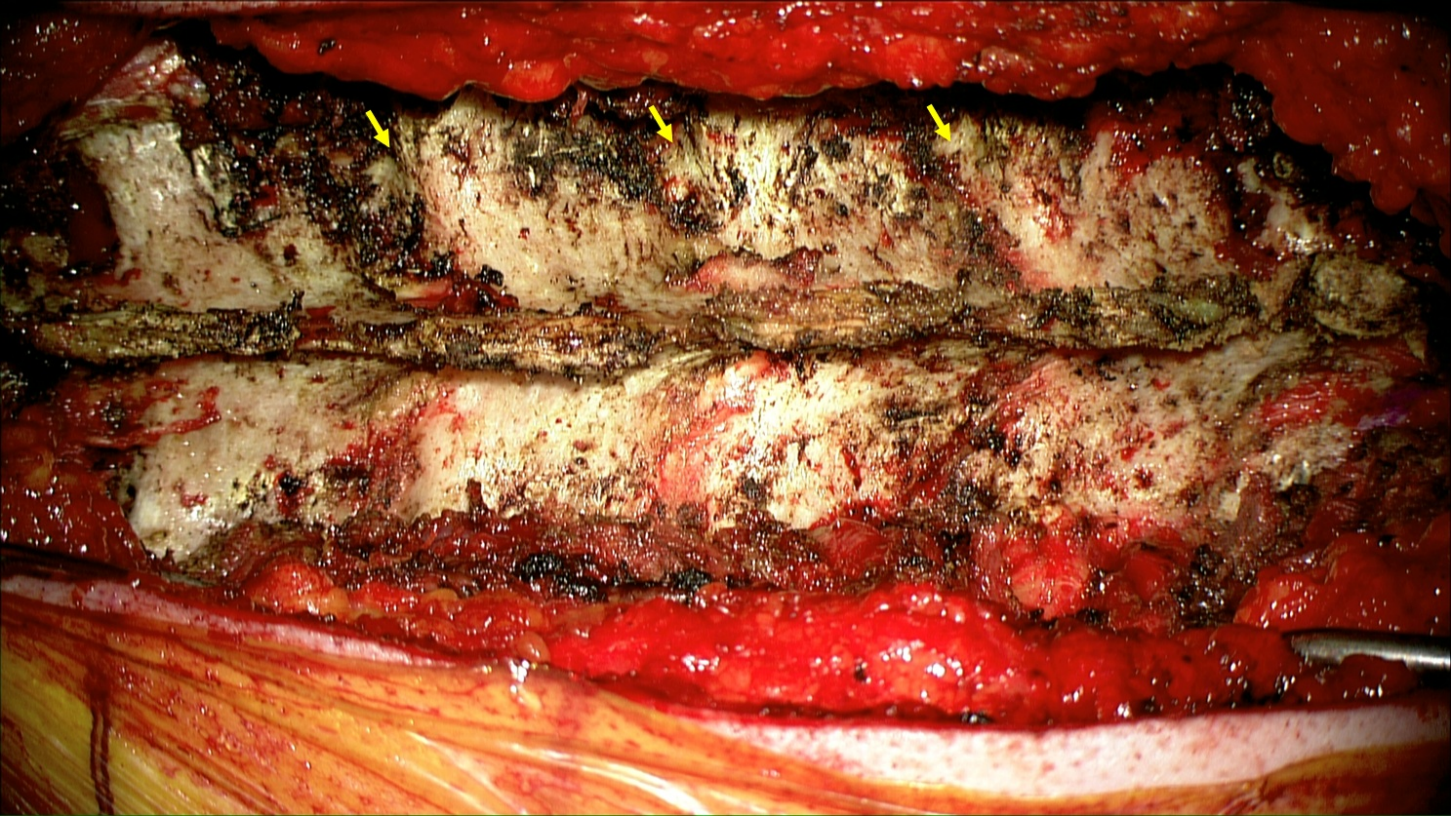
Exposure of thoracic spine showing the lateral edge of the superior articulating process and the transverse process.

After the incision, dissection of the subcutaneous tissue and muscles the thoracic spine is exposed. The surgical exposure should include the superior articulating processes and transverse processes of the vertebrae. The inferior articulating process can be removed, if needed, for osteotomy but is not mandatory for screw placement alone (Figure 1).

Step 2

**Figure 2:**
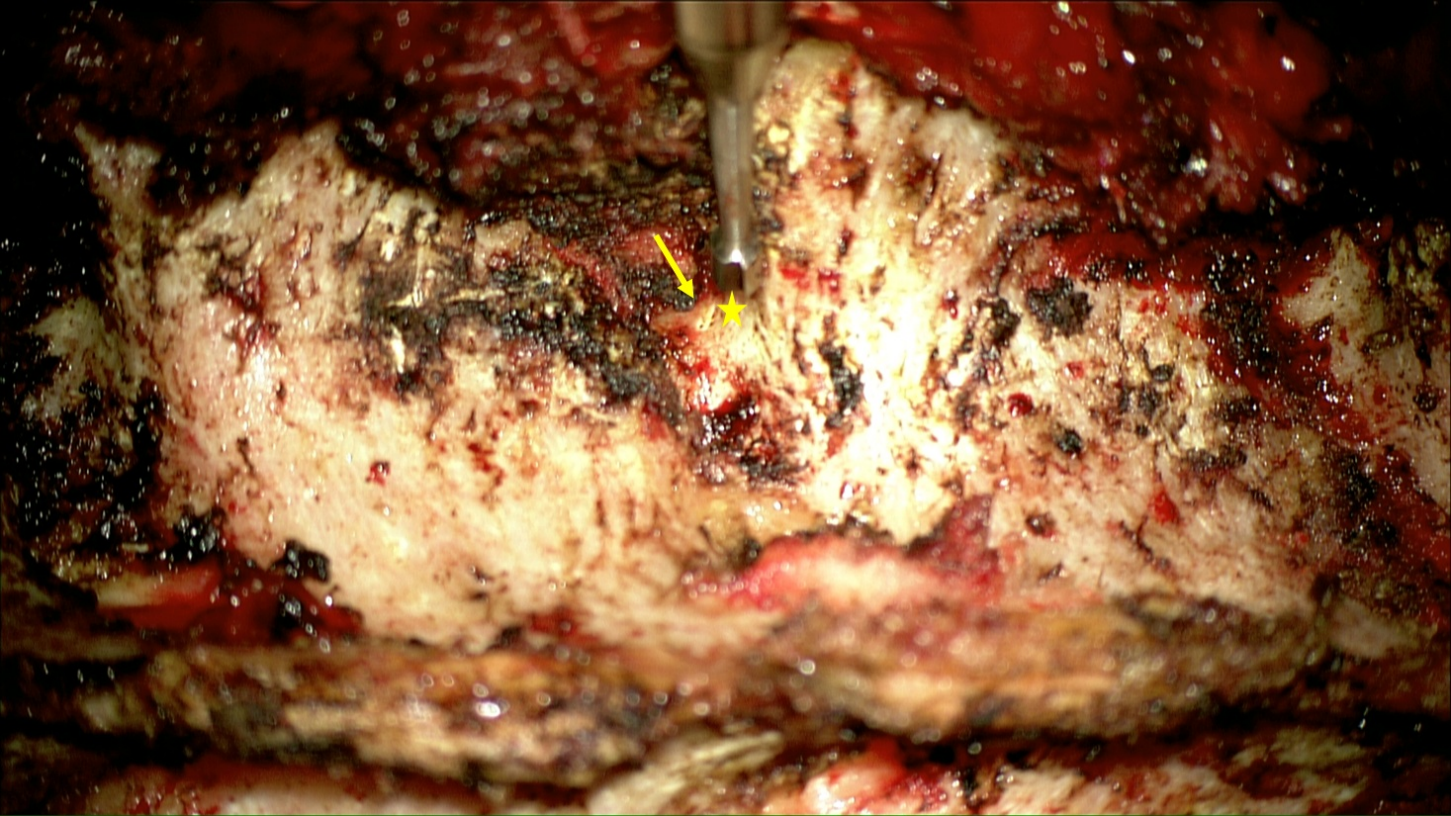
A high-speed drill is used to make an entry point. Note the entry point (star) is just caudal to the lateral edge of the superior articulating process-transverse junction (arrow).

Then, we proceed with a decortication of the bone in the location of the entry point using high-speed electrical or pneumatic drill. For all levels in the thoracic spine, we use a uniform entry point, which is approximately 3 mm caudal to the junction of the lateral margin of the superior articulating process and the transverse process (Figure 2).

Step 3

**Figure 3:**
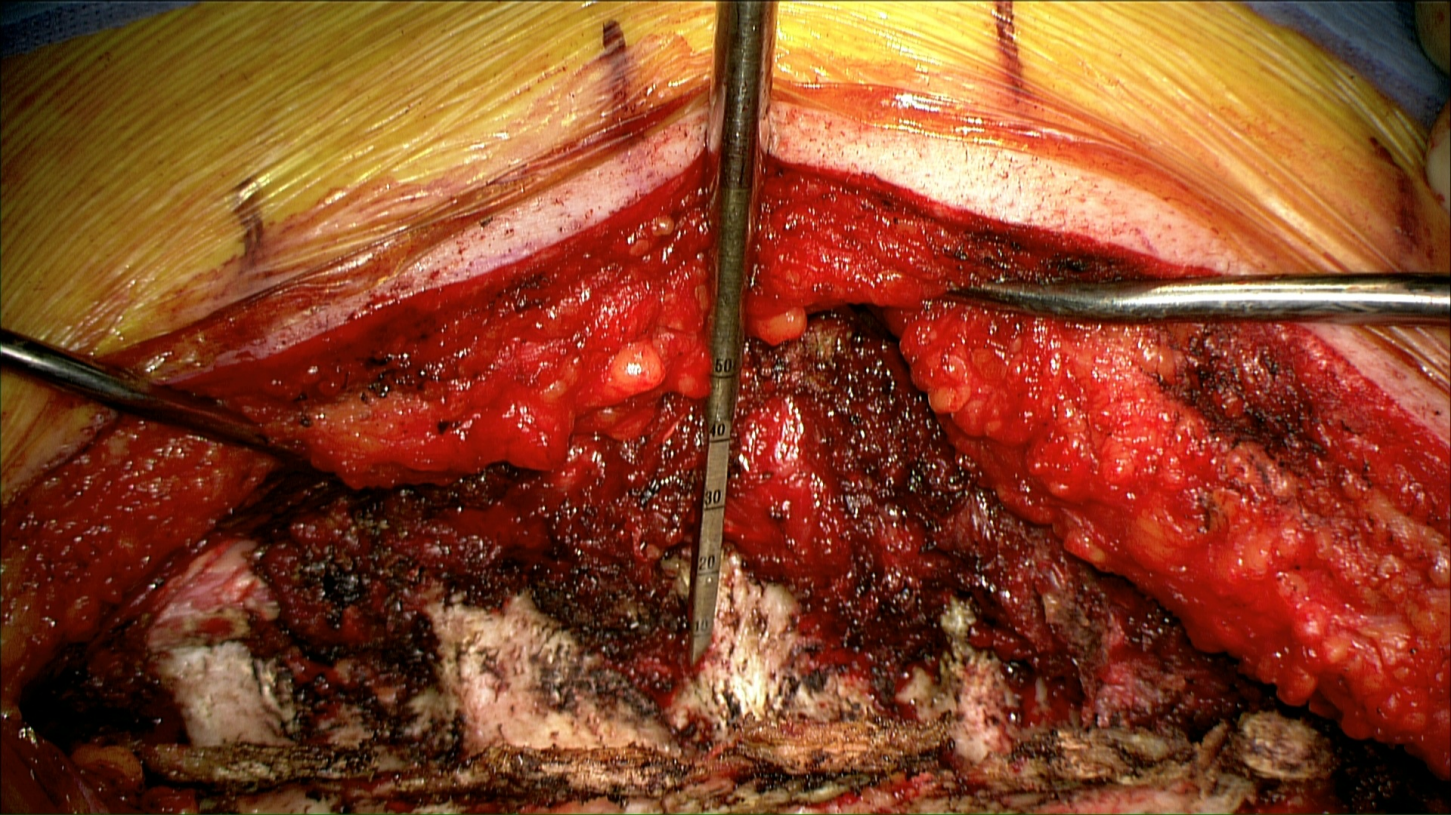
Cannulation of the pedicle using a straight narrow gearshift.

**Figure 4:**
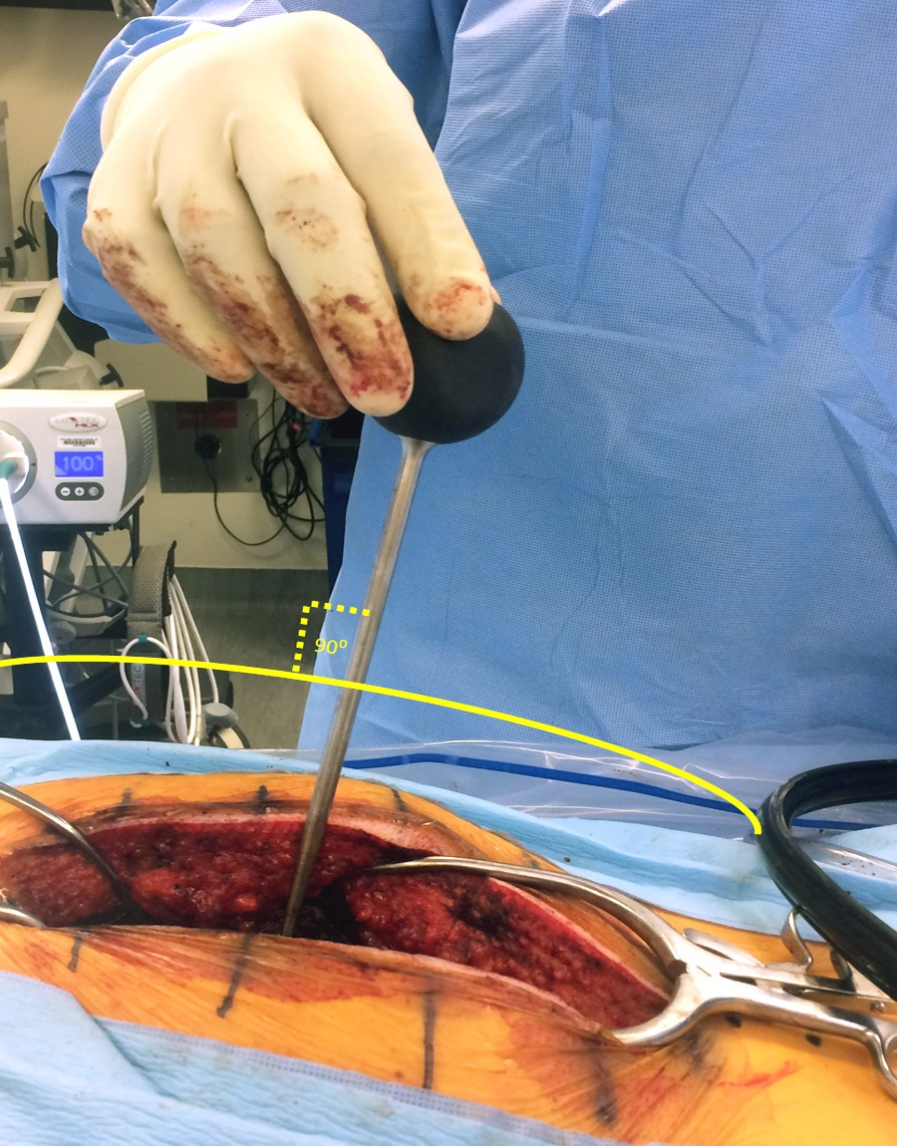
Cannulation is performed in an orthogonal fashion to the dorsal curvature of the spine to ensure a straight trajectory.

The pedicle is cannulated from the entry point and the gearshift probe is further advanced to the desired level based on a preoperative CT analysis, which also determines the length and diameter of the screw to be inserted. A straight gearshift is adequate, and the medial trajectory is approximately 30 degrees for T1 and T2, and 20 degrees for T3 through T12. The pedicle is cannulated in an orthogonal trajectory in relation to the dorsal spine (Figures 3-4).

Step 4

The gearshift probe is removed, and a ball-tipped instrument is inserted to assess for any penetration (breach) of the bony wall in five directions: medially, laterally, superiorly, inferiorly, and anteriorly.

Step 5

With the use of a tap (which is 0.5-1 mm smaller than the intended pedicle screw), we undertap the pedicle tract. Then, the ball-tipped instrument is used to revise any possible vertebral body breach. After this, the pedicle screw can be inserted. Screw insertion can be performed using manual or powered drivers, our preference being the latter for ergonomic ease and better precision.

Step 6

The sagittal trajectory of the pedicle screw insertion should be maintained orthogonal to the curvature of the dorsal spine at each level.

Step 7

C-arm fluoroscopy with anterior-posterior (AP) and lateral views is used for initial localization and subsequent confirmation of appropriate screw insertion. Additional fluoroscopic images with pedicle markers in place are not necessary.                      

**Figure 5:**
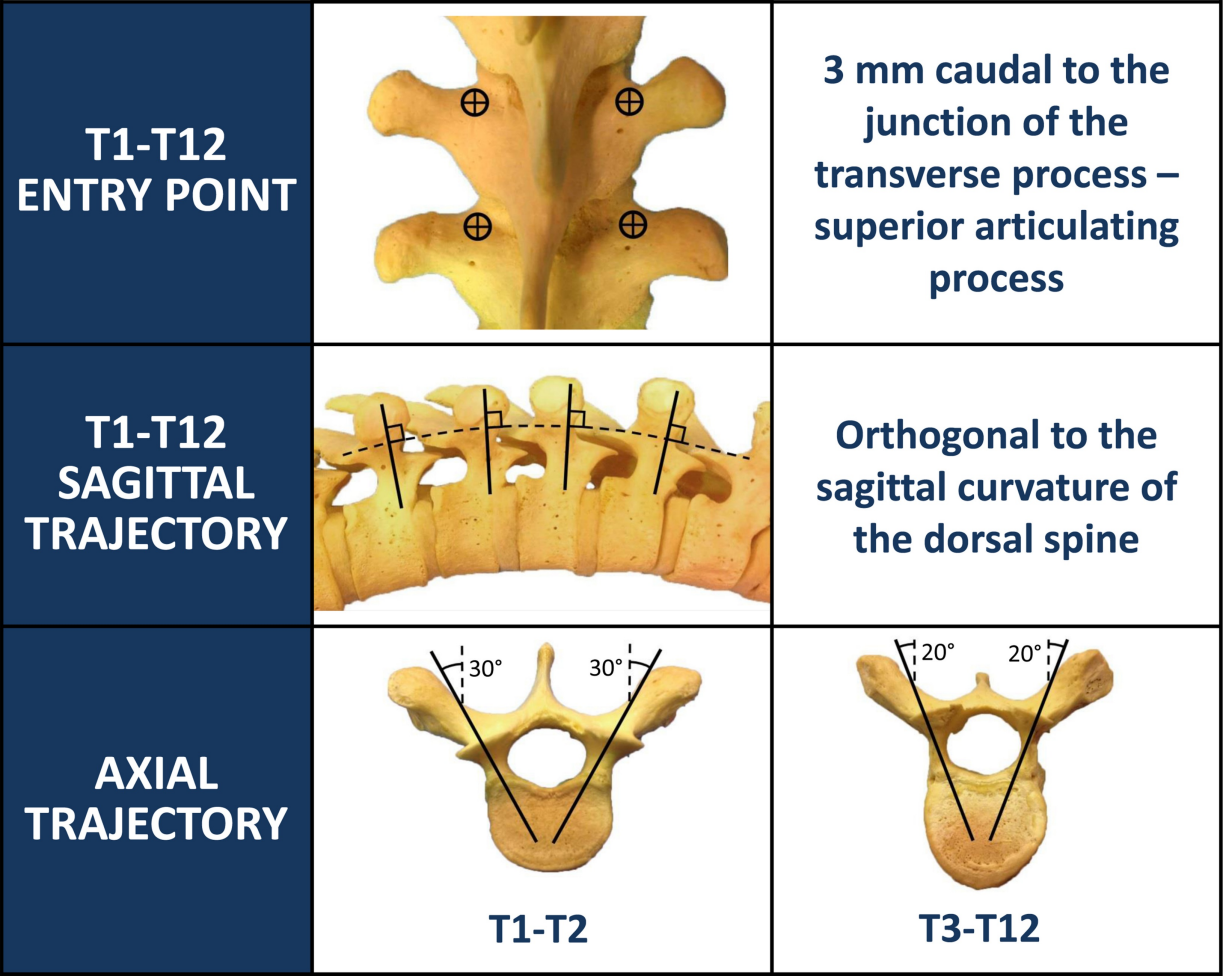
Entry point and trajectory chart for thoracic pedicle screw placement (Copyright: Ali A. Baaj, MD).

**Figure 6:**
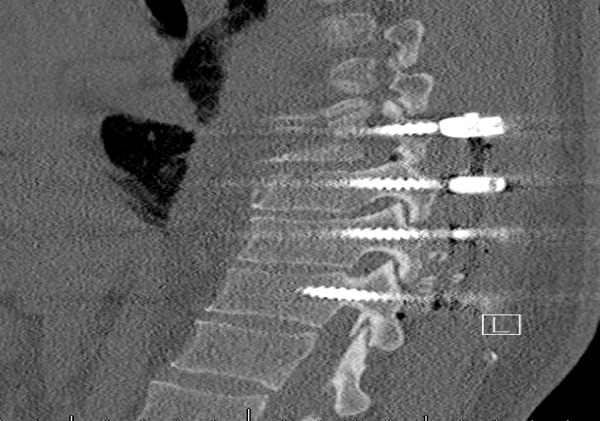
Parasagittal CT demonstrating the position of thoracic screws with proposed entry point and sagittal trajectory.

**Figure 7:**
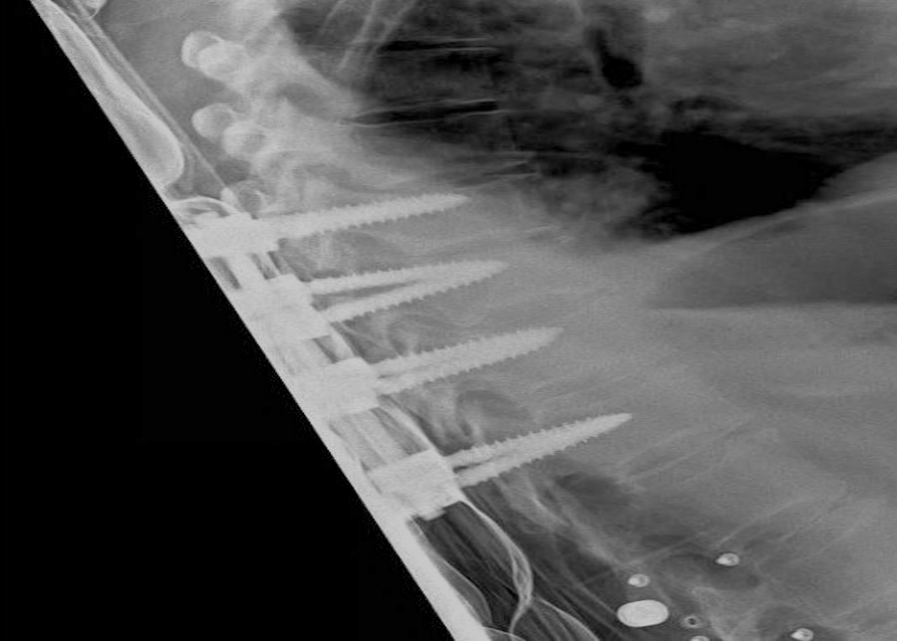
Lateral x-ray image demonstrating trajectories of the pedicle screws.

The uniform entry point that we identified is reproducible at each thoracic level as long as the cranial-caudal orthogonal cannulation trajectory is used as opposed to the earlier studies [2, 8, 21] in which variable entry points were proposed with the tendency to be more lateral and caudal as one proceeds to the upper thoracic region and medial and cephalad as one proceeds distally (Figure 5). Low medial breach rates may be attributed to the importance of the more lateral entry point used in the proposed technique (Figures 6-7). The sagittal trajectory that we use is orthogonal to the curvature of the dorsal spine at the corresponding level. We feel that this is more practical than relating the trajectory to the plane of the transverse processes.

## Conclusions

Freehand thoracic pedicle screw placement is safe and effective and may decrease operative times and radiation exposure. While many of the existing techniques are effective and generally accepted, we have proposed more uniform parameters that could make freehand thoracic pedicle screw placement easier to teach and learn.
